# The Prognostic Significance of Subcarinal Lymph Node Dissection in Esophagectomy for Middle and Lower Thoracic Esophageal Squamous Cell Carcinoma

**DOI:** 10.1093/icvts/ivaf219

**Published:** 2025-09-24

**Authors:** Si Wei Xu, Jun Feng Liu, Yu Rong, Xin Bo Liu, Zhi Hua Shi, Bing Ji Cao, Shao Wei Zhang

**Affiliations:** Department of Thoracic Surgery, Fourth Hospital, Hebei Medical University, Shijiazhuang 050011, China; Department of Thoracic Surgery, Fourth Hospital, Hebei Medical University, Shijiazhuang 050011, China; Department of Thoracic Surgery, Fourth Hospital, Hebei Medical University, Shijiazhuang 050011, China; Department of Thoracic Surgery, Fourth Hospital, Hebei Medical University, Shijiazhuang 050011, China; Department of Thoracic Surgery, Fourth Hospital, Hebei Medical University, Shijiazhuang 050011, China; Department of Thoracic Surgery, Fourth Hospital, Hebei Medical University, Shijiazhuang 050011, China; Department of Thoracic Surgery, Fourth Hospital, Hebei Medical University, Shijiazhuang 050011, China

**Keywords:** esophageal cancer, subcarinal lymph node dissection, prognosis, inverse probability of treatment weighting, esophagectomy

## Abstract

**Objectives:**

This retrospective study assesses the prognostic value of subcarinal lymph node dissection (SCLND) in oesophageal squamous cell carcinoma (ESCC) of the middle and lower thoracic regions.

**Methods:**

The study, conducted at the Fourth Hospital of Hebei Medical University, included 1587 patients with ESCC who underwent radical resection from 2008 to 2014, comprising 204 patients in the non-SCLND group and 1383 patients in the SCLND group. After applying inverse probability of treatment weighting (IPTW) to adjust for confounders, Kaplan-Meier curves, log-rank tests, and Cox regression were used for survival analysis, performed using R.

**Results:**

SCLN metastasis was found in 9.8% of patients. Factors influencing metastasis included pathologic T stage (*P* < .001) and N stage (*P* < .001). SCLN metastasis significantly affected overall survival, with 5-year rates of 49.0% for non-metastatic versus 7.0% for metastatic patients. SCLND improved long-term survival for T3-T4a stage patients but not for T1-T2.

**Conclusions:**

Despite a low SCLN metastasis rate, its presence significantly worsens prognosis. SCLND does not significantly improve long-term survival in patients with pathologic T1-T2 tumuors, but it may confer a survival benefit in T3-T4a stage disease, supporting individualized surgical decisions regarding lymph node dissection.

## Graphical abstract

**Figure:**
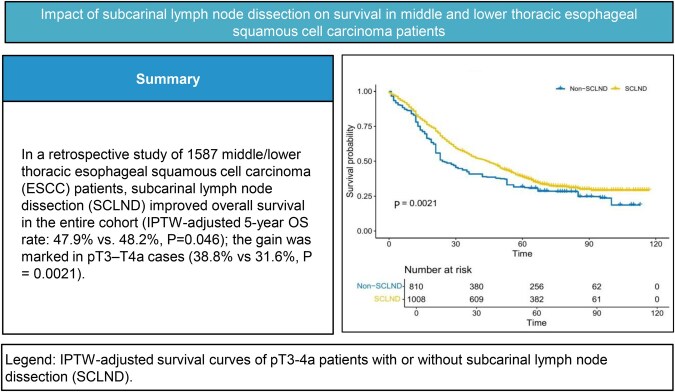

## INTRODUCTION

China is a high-incidence country for oesophageal cancer, with the number of annual cases accounting for approximately half of the global incidence of oesophageal cancer.^1^ Squamous cell carcinoma (SCC) is the most common pathological type, and most patients are diagnosed in the advanced stages, often when seeking medical attention.^2^ The main treatment method at this stage is surgery, which has the potential to offer a cure for patients with resectable oesophageal cancer. However, the prognosis of patients after surgery is poor, and some patients may have lymph node metastasis early after surgery. Lymph node dissection, an essential component of oesophageal cancer surgery, can enhance the accuracy of N staging and potentially improve the prognosis if performed reasonably. However, excessive lymph node dissection not only fails to improve patient outcomes but may heighten the risk of complications, especially pulmonary complications.^3^

The subcarinal lymph node (（SCLN) is located below the bifurcation of the trachea and receives direct ascending lymphatic drainage from the middle and lower oesophagus. It is connected to the paratracheal lymphatic chain and the thoracic duct.^4^ The SCLN, being classified as a regional lymph node for oesophageal cancer and considered crucial for cancer in this region,^5^ necessitates its dissection during oesophagectomy as a routine step. However, there are significant discrepancies in the research results regarding the rate of metastasis to the SCLN, and there is limited research on the impact of dissecting the SCLN on the prognosis of oesophageal cancer patients.^3^^,^^6^^,^^7^

Therefore, this retrospective study used inverse probability of treatment weighting (IPTW) analysis to analyse the influencing factors of SCLN involvement in middle and lower oesophageal cancer, as well as the impact of SCLN resection on prognosis. The aim was to establish a protocol for lymph node dissection during surgery for middle and lower oesophageal cancer, in order to avoid the complications associated with excessive lymph node dissection and the adverse effects of inadequate lymph node staging and prognosis caused by insufficient lymph node dissection.

## METHODS

### Inclusion criteria

Our inclusion criteria were as follows: (1) patients diagnosed with SCC of the middle and lower thoracic oesophagus postoperatively; (2) having undergone resection for oesophageal cancer; (3) intraoperative lymph node dissection of at least 7 nodes; (4) postoperative adjuvant therapy including standard chemotherapy, radiotherapy, or chemoradiotherapy. The postoperative adjuvant therapy regimen consisted of platinum-based double-drug chemotherapy for at least 2 courses and/or radiotherapy for at least one course; (5) no preoperative adjuvant therapy (radiotherapy, chemotherapy, or chemoradiotherapy); and (6) no history of other tumour types. This study was approved by the Ethics Committee of the Fourth Hospital of Hebei Medical University (Approval number: HEBMU-4-IRB-2023KY130). As it was a retrospective study, informed consent was waived.

### Exclusion criteria

This study excluded (1) patients with upper thoracic oesophageal cancer; (2) patients with non-SCC postoperatively; (3) those with lymph node dissection of less than or equal to 6 nodes; and (4) patients at M1 stage.

### Patients

This study collected information on 1587 patients who underwent radical resection for oesophageal cancer at the Fourth Hospital of Hebei Medical University, China, from January 2008 to January 2014. All patients had complete clinical and pathological data. The patients were staged following the 8th edition of the American Joint Committee on Cancer (AJCC)/The Union for International Cancer Control (UICC) TNM classification system for oesophageal cancer.^8^

A total of 1587 patients with complete clinical and follow-up data were included in this study. Among them, 1383 patients underwent subcarinal lymph node dissection (SCLND), and 204 did not. To minimize baseline imbalances and confounding, IPTW was applied using propensity scores derived from 13 covariates (**Table 1**). After weighting, all 1587 patients were retained for survival analysis.

**Table 1.** Clinicopathologic Features Before and After IPTW

Variates	Before IPTW					
	SCLND *n* = 1383	Non-SCLD *n* = 204	SMD	SCLND *n* = 1587.2	Non-SCLD *n* = 1359.9	SMD
Sex						
Female	421	56	0.066	477.7	425.9	0.026
Male	962	148		1109.5	934.0	
Age						
<60	601	89	0.003	691.8	621.3	0.042
≥60	782	115		895.3	738.7	
Smoking						
No	691	92	0.099	784.6	695.0	0.033
Yes	692	112		802.6	665.0	
Drinking						
No	920	92	0.028	1053.7	927.9	0.039
Yes	463	112		533.4	432.0	
Tumour site						
Middle	1064	114	0.457	1177.7	954.1	0.09
Lower	319	90		409.5	405.8	
Length of tumour						
<4 cm	428	76	0.133	504.1	454.9	0.036
≥4 cm	955	128		1083.1	905.1	
Tumour stump						
Negative	1305	185	0.140	1490.9	1274.6	0.009
Positive	78	19		96.3	85.3	
T stage						
T1	223	36	0.159	272.4	269.9	0.074
T2	276	33		309.0	252.4	
T3	819	120		938.6	788.2	
T4a	65	15		67.1	49.4	
N stage						
N0	909	128	0.091	1039.3	924.2	0.076
N1	290	44		333.7	278.0	
N2	152	28		178.5	138.0	
N3	32	4		35.7	19.7	
Adjuvant therapy						
No	642	72	0.228	713.2	567.7	0.064
Yes	741	132		873.9	792.2	
Lymph nodes dissected						
＜15	874	177	0.565	1051.3	1050.4	0.246
≥15	509	27		535.9	309.5	
Differentiation						
Well and moderate	1083	165	0.064	1247.3	1065.5	0.006
Poor	300	39		339.9	294.5	
Vascular tumour thrombus						
No	1340	197	0.018	1537.2	1309.0	0.033
Yes	43	7		50.0	51.0	

### Preoperative staging diagnosis

Upper gastrointestinal endoscopy was performed to assess oesophageal strictures caused by cancer, lesion length, and risk of perforation. Gastroscopy helped to observe the length and circumferential invasion of the tumour and determine the pathological type through biopsy. CT was used to determine the tumour location, size, and its relationship with the surrounding structure as well as the presence of enlarged lymph nodes. B-mode ultrasonography was performed to detect enlarged supraclavicular lymph nodes, and if the short diameter of the lymph nodes exceeded 0.8 cm, needle biopsy was used to exclude metastasis. For early cases, endoscopic ultrasonography is often performed to distinguish them from locally advanced cases; for cases suspected of distant metastasis, PET-CT examination was often conducted for confirmation.

### Surgery

All patients underwent oesophagectomy under double-lumen intubation and intravenous-inhalation combined general anaesthesia. The approach for oesophagectomy was selected as either a left or right thoracotomy based on the location of the cancer. The tubed stomach was used as an oesophageal substitute. For lower thoracic oesophageal cancer, radical oesophagectomy, lymph node dissection, mobilization of the stomach, and anastomosis in the thorax were all completed through a left thoracotomy (Sweet operation). For cancer located in the middle third of the oesophagus, the tubed stomach was constructed through a midline incision in the abdomen, and then radical resection of the oesophagus and lymph nodes, followed by anastomosis in the upper thorax, were performed through a right thoracotomy (Ivor-Lewis oesophagectomy) or in the neck (McKeown operation). Some Ivor-Lewis and McKeown operations were completed using minimally invasive oesophagectomy (MIE) techniques.

### Postoperative adjuvant therapy

Postoperative adjuvant therapy adhered to the guidelines set by the Chinese Society of Clinical Oncology (CSCO), which recommended chemoradiotherapy for patients with T4aN0M0 and T1-4aN + M0 staging.^9^ The administration of chemotherapy and radiotherapy could be either sequential or synchronous, depending on the patient’s tolerability.

Typically, chemotherapy was administered for 3-4 cycles after surgery, and included regimens such as paclitaxel and platinum (TP) or fluorouracil and platinum (FP). For the TP regimen, paclitaxel (China Shiyao Pharmaceutical Group Co., Ltd, Shijiazhuang, China) in a dose of 175 mg/m^2^ (days 1-2) and cisplatin (Qilu Pharmaceutical Group Co., Ltd, Jinan, China) in a dose of 15 mg/m^2^ (days 1-5) were administered intravenously, with a repeat cycle after 3 weeks. For the FP regimen, 5-fluorouracil in a dose of 75 mg/m^2^ (days 1-5) and cisplatin (Qilu Pharmaceutical Group Co., Ltd, Jinan, China) in a dose of 15 mg/m^2^ (days 1-5) were administered intravenously, with a repeat cycle after 3 weeks.

The postoperative radiotherapy field encompassed the tumour bed and high-risk lymphatic drainage area, with a total dose of 54 Gy, delivered at 2.0 Gy per day, 5 times per week.

### Follow-up study

The follow-up study was conducted through phone interviews, email communication, outpatient clinic visits, and utilizing the hospital’s follow-up system. The follow-up assessments included evaluations of clinical symptoms, signs, CT scans, gastroscopy, and pathological biopsy to determine postoperative recurrence and/or metastasis. Follow-up assessments were conducted every six months until the date of death or February 5, 2019. Patients lost to follow-up were censored at the date of first loss to follow-up when 2 consecutive losses to follow-up occurred. This approach ensures that all available data are used without making assumptions about the outcomes of these patients. Given the low number of cases lost to follow-up, we elected not to perform multiple imputation to avoid potential biases that could arise from overfitting the model with limited data. Overall survival (OS) was defined as the time between the date of surgery and either the date of death or the last date of follow-up.

### Statistical analysis

Statistical analyses were conducted using R software version 4.1.2 (R Foundation for Statistical CoFmputing). IPTW was employed to mitigate confounding and baseline imbalances across multiple analyses. Propensity scores (PS) were derived from logistic regression models incorporating 13 baseline covariates: sex, age, tumour location, tumour length, T stage, N stage, tumour differentiation, total number of dissected lymph nodes, smoking history, alcohol consumption history, positive tumour margin, vascular tumour thrombus, and receipt of postoperative adjuvant therapy. Separate PS models were constructed for 3 distinct exposures: (1) presence of SCLN metastasis, (2) receipt of SCLND, and (3) subgroup analyses stratified by T stage (T1, T2, T3-T4a). For each model, IPTW weights were calculated as 1/PS for exposed individuals and 1/(1 − PS) for unexposed individuals. These weights were stabilized and truncated at the 1st and 99th percentiles to minimize the influence of extreme values.

Covariate balance before and after IPTW application was evaluated using standardized mean differences (SMD), with SMD <0.1 considered indicative of adequate balance. Categorical variables (eg, complications) were compared using *χ*^2^ or Fisher’s exact tests, as appropriate. For survival analysis, OS was assessed using IPTW-weighted Kaplan-Meier curves with weighted log-rank tests for univariable comparisons. Multivariable analyses employed weighted Cox proportional hazards models utilizing robust sandwich variance estimators. Within the T stage subgroup analyses, separate IPTW models were fitted for each stratum, excluding T stage as a covariate. The median follow-up duration was estimated using the inverse Kaplan-Meier method. Statistical significance was defined as a 2-sided *P*-value <.05.

## RESULTS

### Postoperative complications

Among 1587 patients, 1383 underwent lymph node dissection and 204 did not; incidences of respiratory failure (2.9% vs 3.4%, *P* = .671), anastomotic leak (4.0% vs 2.0%, *P* = .155), pneumonia (2.2% vs 0.5%, *P* = .126), pulmonary embolism (0.2% vs 0.5%, *P* = .467), vocal-cord paralysis (0.2% vs 0%), chylothorax (0.3% vs 0.5%, *P* = .634), and death (0.2% vs 0.5%, *P* = .467) were numerically higher or equal in the dissection group but not statistically significant, whereas pleural effusion requiring secondary drainage occurred significantly more often after dissection (7.7% vs 3.4%, *P* = .028).

### SCLN metastasis rate

A total of 1587 patients were included in the study, with 1383 patients undergoing SCLND and 136 patients with metastasis of the node, resulting in a SCLN metastasis rate of 9.8%.

### Factors affecting SCLN metastasis

In the univariable logistic regression analysis, drinking history (OR = 1.49, 95% CI: 1.04-2.14, *P* = .029), tumour length ≥4 cm (OR = 2.65, 95% CI: 1.68-4.40, *P* < .001), higher T stage (OR = 3.53, 95% CI: 2.55-5.03, *P* < .001), higher N stage (OR = 7.39, 95% CI: 5.70-9.78, *P* < .001), and vascular tumour thrombus (OR = 6.02, 95% CI: 3.10-11.40, *P* < .001) were significantly associated with SCLN metastasis. Other variables, including smoking history, tumour site, sex, age ≥60 years, and tumour differentiation, were not significantly associated with SCLN metastasis.

In the multivariable logistic regression analysis, T stage (OR = 2.31, 95% CI: 1.53-3.61, *P* < .001) and N stage (OR = 6.49, 95% CI: 4.96-8.67, *P* < .001) remained independent predictors of SCLN metastasis. Smoking, drinking, tumour length, and vascular tumour thrombus were not statistically significant after adjustment.

### Factors affecting overall survival

A total of1587 patients were enrolled, and as of the follow-up date, 926 patients were alive and 661 patients had died. The MFT was 76 months (95% CI: 75.0-77.0 months) in the entire cohort. Of the1587 patients, 116 (7.3%) were lost to follow-up. These patients had 2 consecutive losses and were imputed as deceased. In the SCLND group, the MFT was 76 months (95% CI: 75.0-77.0 months), while in the non-SCLND group, it was 80 months (95% CI: 79.0-85.0 months). The 3-year and 5-year OS rates were 63.3% and 47.8%, respectively. The median survival time was 57 months (range: 1-117 months).

### Univariable analysis for OS

IPTW-weighted univariable Cox regression revealed that sex (*P* = .002), smoking (*P* = .005), alcohol consumption (*P* = .024), tumour length ≥4 cm (*P* < .001), higher T stage (*P* < .001), higher N stage (*P* < .001), positive tumour stump margins (*P* = .002), vascular tumour thrombus (*P* = .003), receipt of adjuvant therapy (*P* < .001), and fewer than 15 dissected lymph nodes (*P* < .001) were significantly associated with OS. In contrast, age (<60 vs ≥60, *P* = .765), tumour site (middle vs lower, *P* = .315), and differentiation (well/moderate vs poor, *P* = .394) were not statistically significant (**Table 2**).

**Table 2.** The IPTW-Weighted Univariable and Multivariable Cox Regression Analysis for Overall Survival (OS) in Oesophageal Cancer

	Univariable analysis		Multivariable analysis	
	HR (95% CI)	*P*	HR (95% CI)	*P*
Sex (M vs F)	1.46 (1.15-1.86)	0.002	1.10 (0.81-1.49)	0.545
Age (＜60 vs ≥60)	0.97 (0.79-1.19)	0.765	1.05 (0.87-1.26)	0.622
Smoking (Yes vs No)	1.35 (1.1-1.65)	0.005	1.07 (0.81-1.42)	0.637
Drinking (Yes vs No)	1.27 (1.03-1.56)	0.024	1.04 (0.82-1.32)	0.745
Site of tumour (M vs L)	1.12 (0.9-1.39)	0.315	–	–
Length of tumour (≥4 cm vs ＜4 cm)	1.78 (1.42-2.25)	<0.001	1.29 (1.02-1.63)	0.033
T2	1.63 (1.09-2.45)	0.018	1.67 (1.14-2.45)	0.009
T3	3.47 (2.52-4.78)	<0.001	2.82 (2.04-3.90)	<0.001
T4	3.89 (2.46-6.15)	<0.001	2.40 (1.45-3.97)	<0.001
N1	2.42 (1.88-3.1)	<0.001	2.12 (1.70-2.65)	<0.001
N2	4.05 (3.17-5.18)	<0.001	3.06 (2.37-3.94)	<0.001
N3	5.26 (3.25-8.53)	<0.001	3.37 (1.84-6.16)	<0.001
Adjuvant therapy (No vs Yes)	1.68 (1.36-2.08)	<0.001	2.15 (1.78-2.61)	<0.001
Differentiation (Well + Med vs Poor)	1.12 (0.86-1.46)	0.394	–	–
LNs dissected (＜15 vs ≥ 15)	0.58 (0.45-0.74)	<0.001	0.63 (0.50-0.79)	<0.001
Stump (positive vs negative)	1.62 (1.19-2.21)	0.002	1.09 (0.76-1.56)	0.651
Vascular tumour thrombus (Yes vs No）	2.19 (1.31-3.63)	0.003	1.31 (0.84-2.03)	0.231

### Multivariable analysis for OS

In the IPTW-weighted multivariable Cox regression model, higher T stage (*P* < .001), higher N stage (*P* < .001), tumour length ≥4 cm (*P* = .033), absence of adjuvant therapy (*P* < .001), and fewer than 15 dissected lymph nodes (*P* < .001) were identified as independent predictors of OS (**Table 2**).

### The impact of SCLN metastasis on OS

**Figure 1.**
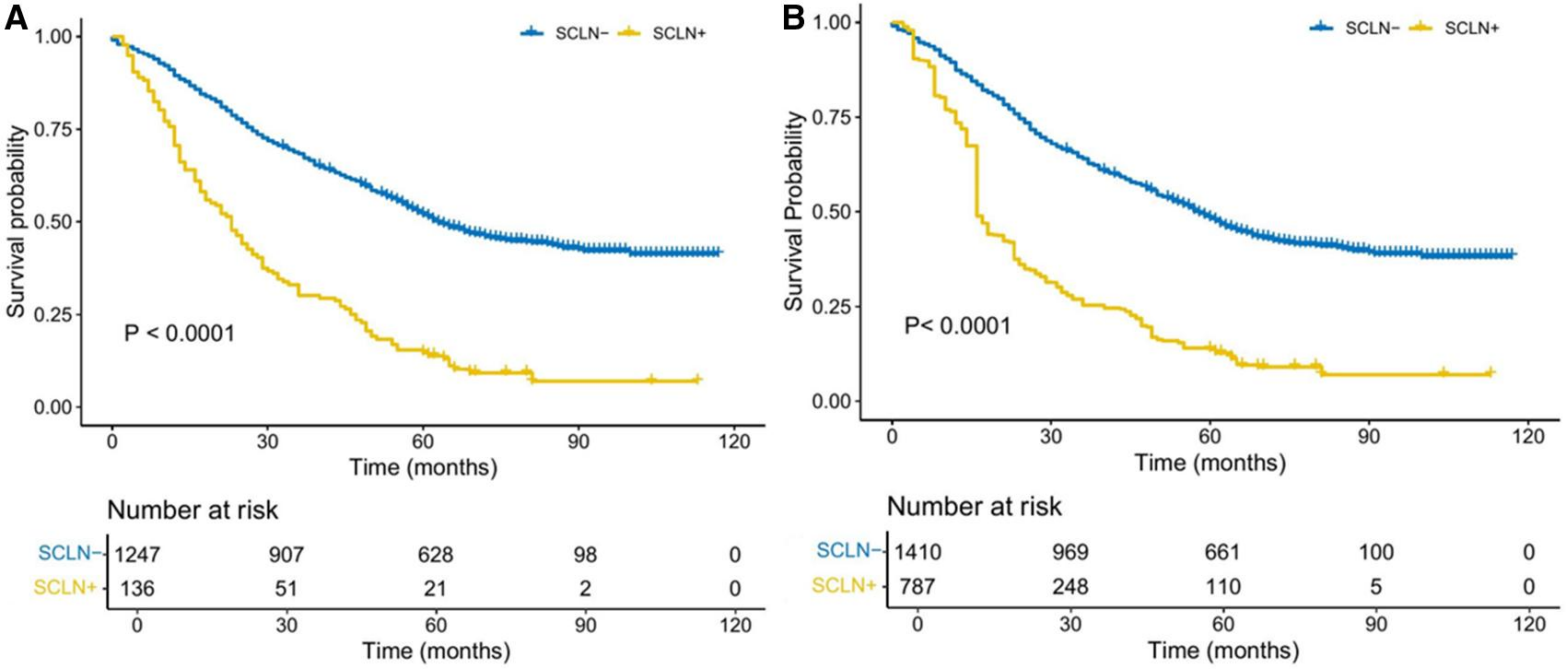
Kaplan-Meier Estimates of Overall Survival Before (A) and After (B) Inverse Probability of Treatment Weighting (IPTW). (A) Pre-IPTW curves show markedly worse survival for patients with SCLN metastasis compared with those without. (B) Following IPTW adjustment, the survival gap persists, confirming that SCLN metastasis remains an independent predictor of poorer prognosis

After IPTW adjustment, patients with SCLN metastasis had significantly poorer OS compared to those without metastasis. The 5-year survival rate was 7.0% in the SCLN-positive group versus 49.0% in the SCLN-negative group. This difference remained statistically significant in the IPTW-weighted Kaplan-Meier analysis (*P* < .001), highlighting the adverse prognostic impact of SCLN involvement (**Figure 1**).

### The impact of SCLND on OS

**Figure 2.**
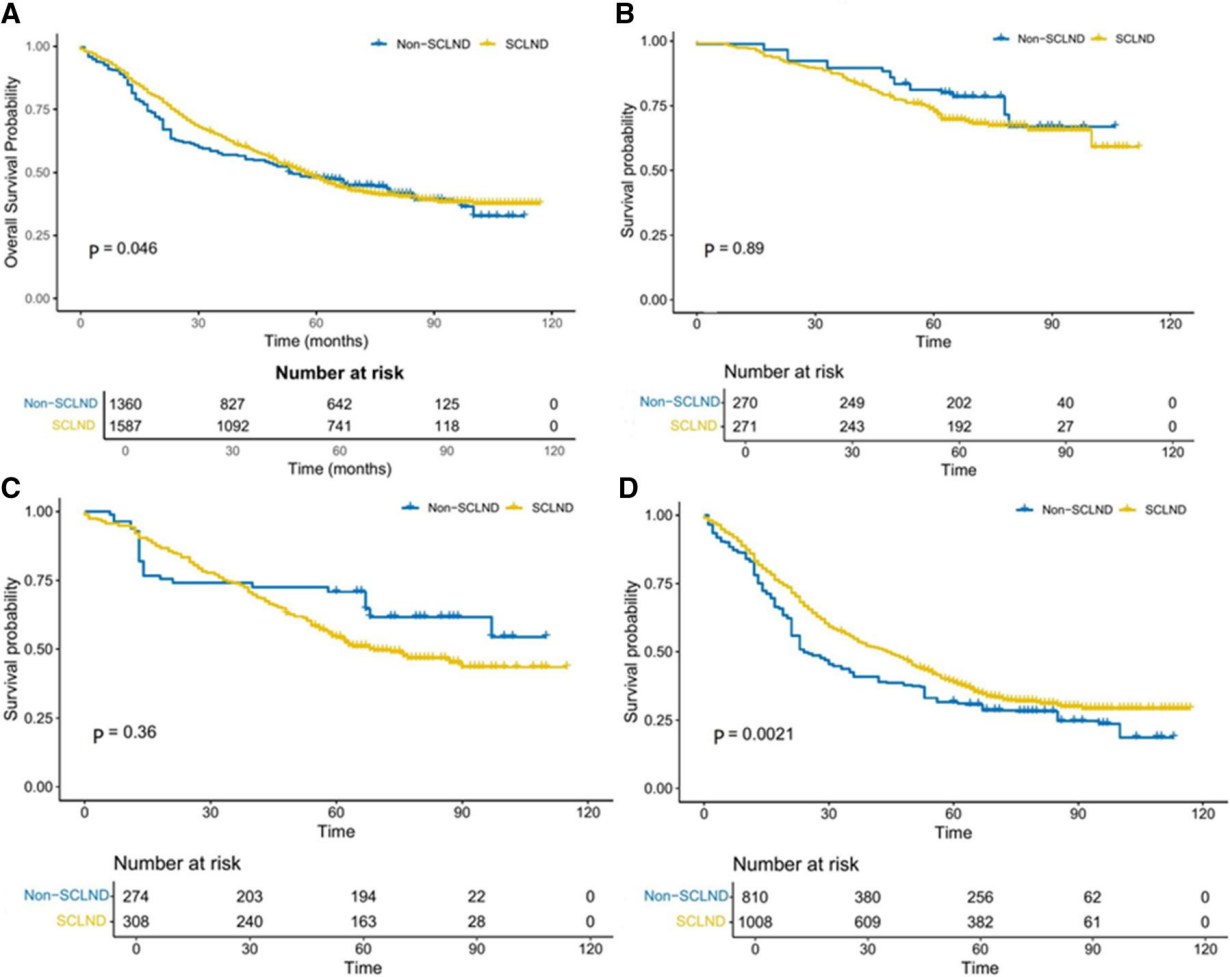
IPTW-Adjusted Kaplan-Meier Overall Survival Curves by SCLND Status. (A) Entire cohort: survival curves for the SCLND and non-SCLND groups overlap (*P* > .05). (B) T1 stage: no survival advantage with SCLND (*P* > .05). (C) T2 stage: no survival advantage with SCLND (*P* > .05). (D) T3-4a stage: SCLND conferred a marked survival benefit compared with non-SCLND (*P* < .05)

The impact of SCLND on OS was evaluated using IPTW-weighted Kaplan-Meier analysis. After adjustment, the 5-year OS rate was 47.9% in the SCLND group and 48.2% in the non-SCLND group, a difference that was statistically significant (*P* = .046) (**Figure 2A**). In the T1 subgroup, the 5-year OS was 73.1% in the SCLND group versus 81.1% in the non-SCLND group (*P* = .89), while in the T2 subgroup, it was 54.4% versus 70.8%, respectively (*P* = .36); neither difference reached statistical significance (**Figure 2B and C**). However, in patients with advanced-stage tumuors (T3-T4a), SCLND was associated with improved survival: the 5-year OS rate was 38.8% in the SCLND group versus 31.6% in the non-SCLND group (*P* = .0021) (**Figure 2D**).

## DISCUSSION

Lymph node metastasis is a critical aspect of oesophageal cancer progression and is associated with a less favourable prognosis. The oesophageal submucosa contains a dense longitudinal lymphatic network that facilitates lymph node skipping metastasis in early oesophageal cancer. Conversely, the oesophageal muscularis contains a transverse lymphatic system that drains into periesophageal lymph nodes or connects directly with the paratracheal lymphatic chain or thoracic duct. The SCLN plays a pivotal role in the lymphatic drainage of middle and lower oesophageal cancer.^4^

Lymph node dissection is a crucial component of oesophageal cancer surgery, as it ensures more precise postoperative pathological staging and may enhance long-term survival when performed appropriately. However, overzealous dissection can lead to postoperative complications, such as increased pneumonia risk due to SCLN dissection.^3^ In this study, although there was no increase in major postoperative complications, additional SCLND showed a trend of increasing pleural effusion, thereby increasing drainage volume. Thus, understanding the metastatic status of SCLNs and their impact on long-term survival is essential.

The metastatic rate of SCLNs is relatively low, ranging from 7.0% to 22.9%.^6^^,^^7^^,^^10^ Several studies have evaluated risk factors for subcarinal metastasis. Niwa et al reported a 7.0% SCLN metastasis rate in thoracic oesophageal cancer, with clinical T stage (T2-T4) being an independent predictor of pathological SCLN metastasis (*P* = .021).^6^ Feng et al found a 22.9% subcarinal LN metastasis rate, with factors such as tumour length (>3 cm), tumour location (lower vs upper/middle), vessel involvement, and depth of invasion associated with increased SCLN metastasis risk.^7^ In this study, the SCLN metastasis rate was 10.3%, strongly associated with T stage (*P* < .0001). As invasion depth increased, the metastasis rate also increased (*P* < .0001). These findings suggest that SCLN metastasis is influenced by invasion depth and may not be necessary for patients with T1 and T2 oesophageal cancer. However, further research is needed to validate these conclusions, particularly regarding long-term survival implications.

SCLN metastasis is rare in upper thoracic oesophageal cancer. Shibamoto et al reported a 0% SCLN metastasis rate from upper thoracic oesophageal cancer,^11^ while Shang et al found an 8.6% rate, lower than that from middle (19.1%) and lower (16.2%) thoracic oesophageal cancer.^12^ Therefore, SCLN dissection is not recommended for upper thoracic oesophageal cancer.

Lymph node involvement is a significant prognostic factor in oesophageal cancer. Feng et al reported that patients with SCLN metastasis exhibited a markedly reduced 5-year survival rate compared to those without metastasis (26.7% vs 60.9%; *P* < .0001).^7^ Similarly, Niwa et al found that patients with pathological SCLN metastasis demonstrated a significantly decreased 5-year disease-free survival rate (23.1% vs 67.5%; log-rank *P* < .0001).^6^ In this study, the 5-year survival rate for patients with SCLN metastasis was 7.0% compared to 49.0% in those without (*P* < .0001). These findings consistently indicate that SCLN metastasis negatively impacts patient prognosis.

The impact of SCLND on long-term survival remains unclear. Hu et al found no significant difference in 5-year survival rates between the non-dissection and dissection groups (38.9% vs 34.3%; *P* > 0.05).^3^ Niwa et al suggested that SCLND may not provide significant value for upper and lower thoracic ESCC patients, particularly in superficial carcinoma cases.^6^ Shang et al proposed omitting SCLND for upper thoracic ESCC patients but recommended it for middle and lower thoracic cases.^12^ This study stratified middle and lower oesophageal cancer patients by T staging and found that SCLND had no significant impact on T1-2 patients but significantly improved survival in T3-4a patients or the overall population. Thus, SCLND is recommended for T3-4a patients but can be omitted for T1-2 patients.

Preoperative diagnosis of SCLN metastasis remains challenging. While endobronchial ultrasound-guided transbronchial needle aspiration (EBUS-TBNA) is theoretically viable, limited research exists. Positron emission tomography/CT (PET/CT) exhibits high specificity but is limited by suboptimal sensitivity and cost.^13^ Therefore, preoperative diagnosis requires integrating various imaging modalities such as CT, endoscopic ultrasound (EUS), and PET/CT.

This study used IPTW to balance baseline characteristics between the SCLND and non-SCLND groups, reducing selection bias and confounding effects. IPTW is widely used in observational studies to create comparable groups. Despite challenges in achieving perfect matching due to sample size limitations, the matching process resulted in well-balanced groups with standardized mean differences below 0.10 for key covariates.

Prior research in ICVTS has focused on oesophageal cancer treatments, such as uniportal VATS oesophagectomy, which minimizes invasiveness and improves patient outcomes.^14^ Bertrand et al highlighted the management of neo-oesophagus-airway fistula (NEAF) post-oesophagectomy, suggesting nonoperative management followed by surgery leads to better 1-year survival rates.^15^ This study contributes to the discourse by examining the prognostic significance of SCLND in middle and lower thoracic SCC, offering robust evidence that SCLND impacts long-term survival, particularly for T3-T4a patients. The findings guide surgical decisions regarding lymph node dissection in oesophageal cancer treatment.

However, limitations should be considered: (1) Retrospective design introduces selection and information bias. (2) Single-center data limits diversity and applicability. (3) Follow-up completeness may be affected by loss to follow-up. (4) Variability in surgical techniques may influence outcomes. (5) Residual confounding factors may exist despite IPTW adjustments.

In summary, although thoracic ESCC patients rarely develop SCLN metastasis, its presence markedly worsens prognosis. SCLND offers no survival benefit for T1-T2 disease but significantly improves outcomes in T3-T4a patients.

## Data Availability

Data will be made available upon request to researchers who meet the criteria for access to confidential data.
